# Risk Factors for Death Among 120,804 Hospitalized Patients with Confirmed COVID-19 in São Paulo, Brazil

**DOI:** 10.4269/ajtmh.20-1598

**Published:** 2021-05-31

**Authors:** Patricia Vieira da Silva, Silvano Barbosa de Oliveira, Juan José Cortez Escalante, Maria Almiron, Daniel Henrique Tsuha, Helena Keico Sato, Paulo Rossi Menezes, Regiane Cardoso de Paula, Tatiana Lang D’Agostini, Julio Croda

**Affiliations:** 1 Federal University of Mato Grosso do Sul, Campo Grande, Brazil;; 2 Pan American Health Organization, Brasília, Brazil;; 3 World Health Organization, Brasília, Brazil;; 4 University of Brasilia, Brasília, Brazil;; 5 Epidemiological Surveillance Center “Prof. Alexandre Vranjac” of the São Paulo State Department of Health, São Paulo, Brazil;; 6 Disease Control Coordination of the São Paulo State Department of Health, São Paulo, Brazil;; 7 Oswaldo Cruz Foundation, Campo Grande, Brazil

## Abstract

São Paulo is a state in Brazil with one of the highest numbers of confirmed and severe cases of coronavirus disease (COVID-19), with an incidence of 294 hospitalizations per 100,000 inhabitants. We report the clinical characteristics and outcomes of 120,804 hospitalized patients with confirmed COVID-19 from February 26 to October 10, 2020, in São Paulo. Characteristics of patients who died and survived were compared using a survival analysis. The median age was 60 years (interquartile range [IQR], 47–72), 67,821 (56.1%) were men, and 61,659 (51.0%) were white. Most hospitalized patients (79,812; 66.1%) reported one or more comorbidities, 41,708 (34.5%) hospitalized patients were admitted to intensive care units, and 33,079 (27.4%) died. Men (hazard ratio [HR], 1.22; 95% confidence interval [CI], 1.18–1.25), elderly individuals (HR, 3.85; 95% CI, 3.68–4.02), and patients with chronic cardiovascular disease including hypertension (HR, 1.05; 95% CI, 1.02–1.08), chronic lung disease (HR, 1.38; 95% CI, 1.31–1.45), diabetes mellitus (HR, 1.14; 95% CI, 1.11–1.18), and chronic neurological disease (HR, 1.48; 95% CI, 1.41–1.55) were at higher risk for death from COVID-19.

## BACKGROUND

In December 2019, an outbreak of pneumonia of unknown origin was reported in Wuhan city, Hubei province, China. The etiological agent was subsequently identified as severe acute respiratory syndrome coronavirus 2 (SARS-CoV-2), and the illness was identified as novel coronavirus disease (COVID-19) by the World Health Organization.^1^ In Brazil, the first COVID-19 case was reported in Sao Paulo City in a 61-year-old man whose onset of symptoms began on February 23, 2020.^2^ Since the writing of this report, more than 7 million confirmed cases and 180,000 deaths have been reported in Brazil, with the highest number of hospitalized patients and deaths occurring in the state of São Paulo.^3^

Because COVID-19 is an emerging disease, there is limited knowledge about its clinical characteristics and outcomes among hospitalized patients. Studies have shown that among individuals with confirmed cases of COVID-19, older people and those with underlying clinical conditions are at higher risk for hospitalization and death.^4^ A systematic review conducted in China found that of 2,087 hospitalized patients with cases considered critical, 49% (1,023) died. Of these 1,023 patients, 81% (829 of 1,023) were 60 years or older. When considering the existence of underlying clinical conditions, mortality among patients without comorbidities was much lower (0.9%; 133 of 15,536) than that of patients with one or more comorbidities.^5^ A similar pattern was observed in the United States^4^ and Italy.^6^

Because Latin-America has a different demographic population than China and many countries in Europe, a notable concern is whether different age groups and severity might be observed as the virus spreads among the Brazilian population. During this study, we describe the risk factors for death among 120,804 hospitalized patients with confirmed COVID-19 cases in São Paulo, Brazil, from February 26 to October 10, 2020.

## MATERIALS AND METHODS

This case series included all hospitalized patients with confirmed COVID-19 in the state of São Paulo registered in the Influenza Epidemiological Surveillance System (SIVEP-GRIPE) from February 26, 2020 to October 10, 2020.^7^ The SIVEP-GRIPE is a system used by health professionals and institutions from the public or private sector throughout the national territory to report hospitalized patients with severe acute respiratory illness (SARI) and deaths attributable to SARI regardless of hospitalization. For this analysis, epidemiological, clinical, and outcome data of the hospitalized patients with COVID-19 (confirmed by real-time RT-PCR)^8^ from SIVEP-GRIPE were used.

The criteria for hospitalization and case management according to the guidelines of the World Health Organization (WHO)^8^ and Brazilian Ministry of Health^9^ were followed during the observation period. According to these guidelines, cases of COVID-19 that progressed to SARI were observed in patients who presented with clinical criteria for flu and at least one of the following symptoms: shortness of breath (dyspnea/respiratory distress); sensation of persistent pressure in the chest; O_2_ saturation less than 95% in ambient air; or bluish coloring of the lips or face.

Critical cases were defined as hospitalization with SARI, the need to be admitted to the intensive care unit (ICU) because of the presence of organic disorders or hemodynamic instability, and requiring mechanical ventilation or other intensive care procedures. To qualify for admission to the ICU, it was necessary to present at least one of the following criteria: acute respiratory failure requiring invasive mechanical ventilation; acute respiratory failure with the need for noninvasive ventilation (especially when there is a need for FiO_2_ > 50%, or inspiratory positive airway pressure > 10 cm H_2_O or expiratory positive airway pressure > 10 cm H_2_O to maintain SpO_2_ > 94% and/or respiratory frequency ≤ 24 rpm); PaCO_2_ ≥ 50 mm Hg, and pH ≤ 7.35; or hemodynamic instability or shock defined as arterial hypotension (systolic blood pressure < 90 mmHg or mean arterial pressure < 65 mmHg). The care of these patients with SARI followed the protocols nationally standardized by the Ministry of Health.

The χ^2^ test was used to compare the differences among fatal cases. The Kaplan-Meier survival analysis was performed to analyze the probability of death during the hospitalization of severely ill patients with COVID-19. For the occurrence of death, the observation period was considered from the date of symptom onset until the occurrence of the fatal outcome. When the outcome of death did not occur, October 10 was selected as the final date of observation. Furthermore, the four more prevalent comorbidities among the patients included in the study were analyzed (chronic cardiovascular disease including hypertension, chronic lung disease, diabetes mellitus, and chronic neurological disease). The outcome was adjusted for age, sex, and all comorbidities, as well as their interactions using Cox regression. The survival analysis was performed using three age categories; younger 60 years, 60 to 79 years, and older than 80 years. Statistical analyses were conducted using R software with Survival package.

Although the research did not require approval, the researchers express their ethical commitment to the management, analysis, and publication of data in accordance with Resolution 466/12 and 510/16 of the National Health Council.

## RESULTS

In São Paulo state, from February 26 to October 10, 2020, a total of 120,804 hospitalized patients with laboratory-confirmed COVID-19 were reported in 623 of 645 municipalities, comprising an incidence of 294 hospitalizations per 100,000 inhabitants. The characteristics of patients with COVID-19 during hospitalization and outcomes are summarized in Table 1. The median age of hospitalized patients was 60 years (interquartile range [IQR], 47–72 years), 67,821 (56.1%) were men, and 61,659 (51.0%) were white. Most hospitalized patients (79,812; 66.1%) reported one or more comorbidities; among them, 27,000 (33.8%) died. More than 41,000 hospitalized patients (34.5%) were admitted to ICUs and 33,079 (27.4%) died.

**Table 1:** Characteristics of patients with COVID-19 during hospitalization and outcomes

Characteristics	Total	Dead	Alive	*P* value
	n (%)	n (%)	n (%)	
Total	120,804 (100.0)	33,079 (27.4)	87,725 (72.6)	
Sex				
Male	67,821 (100.0)	19,087 (28.1)	48,734 (71.9)	< 0.001
Female	52,974 (100.0)	13,992 (26.4)	38,982 (73.6)	
Missing	9 (100.0)	0 (0.0)	9 (100.0)	
Age group, y				
< 30	5879 (100.0)	358 (6.1)	5,521 (93.9)	< 0.001
30–59	53,368 (100.0)	7,278 (13.6)	46,090 (86.4)	
≥ 60	61,475 (100.0)	25,415 (41.3)	36,060 (58.7)	
Missing	82 (100.0)	28 (34.1)	54 (65.9)	
Race*				
White	61,659 (100.0)	17,902 (29.0)	43,757 (71.0)	< 0.001
Black	6,382 (100.0)	2,013 (31.5)	4,369 (68.5)	
Yellow	1,500 (100.0)	503 (33.5)	997 (66.5)	
Mixed	23,386 (100.0)	6,567 (28.1)	16,819 (71.9)	
Indigenous	87 (100.0)	19 (21.8)	68 (78.2)	
Missing	27,790 (100.0)	6,075 (21.9)	21,715 (78.1)	
Comorbidity (none, 1, or > 1†)				
Yes	79,812 (100.0)	27,000 (33.8)	52,812 (66.2)	< 0.001
No	224 (100.0)	46 (20.5)	178 (79.5)	
Missing	40,768 (100.0)	6,033 (14.8)	34,735 (85.2)	
Chronic cardiovascular disease + hypertension				
Yes	44,041 (100.0)	16,213 (36.8)	27,828 (63.2)	< 0.001
No	22,187 (100.0)	6,832 (30.8)	15,355 (69.2)	
Missing	54,576 (100.0)	10,034 (18.4)	44,542 (81.6)	
Chronic lung disease				
Yes	4,723 (100.0)	2,252 (47.7)	2,471 (52.3)	< 0.001
No	47,025 (100.0)	15,693 (33.4)	31,332 (66.6)	
Missing	69,056 (100.0)	15,134 (21.9)	53,922 (78.1)	
Diabetes mellitus				
Yes	31,903 (100.0)	11,702 (36.7)	20,201 (63.3)	< 0.001
No	29,847 (100.0)	9,806 (32.9)	20,041 (67.1)	
Missing	59,054 (100.0)	11,571 (19.6)	47,483 (80.4)	
Chronic neurological disease				
Yes	5,580 (100.0)	2,954 (52.9)	2,626 (47.1)	< 0.001
No	46,515 (100.0)	15,261 (32.8)	31,254 (67.2)	
Missing	68,709 (100.0)	14,864 (21.6)	53,845 (78.4)	
ICU				
Yes	41,708 (100.0)	20,220 (48.5)	21,488 (51.5)	< 0.001
No	79,096 (100.0)	12,859 (16.3)	66,237 (83.7)	

Severe cases: patients had been hospitalized in the general ward only. Critical cases: patients have been hospitalized in the ICU. Source: REDCap and Influenza Epidemiological Surveillance System (SIVEP-GRIPE) Epidemiological Surveillance Center of the State Health Secretary of São Paulo (CVE/SES-SP).

* Brazilian Institute of Geography and Statistics (IBGE) classification

† Chronic cardiovascular disease plus hypertension, diabetes mellitus, chronic lung disease, chronic liver disease, chronic hematological disease, Down syndrome, chronic neurological disease, immunodeficiency or immunodepression/HIV, chronic kidney disease, obesity, neoplasm (solid or hematological tumor).

**Figure 1.**
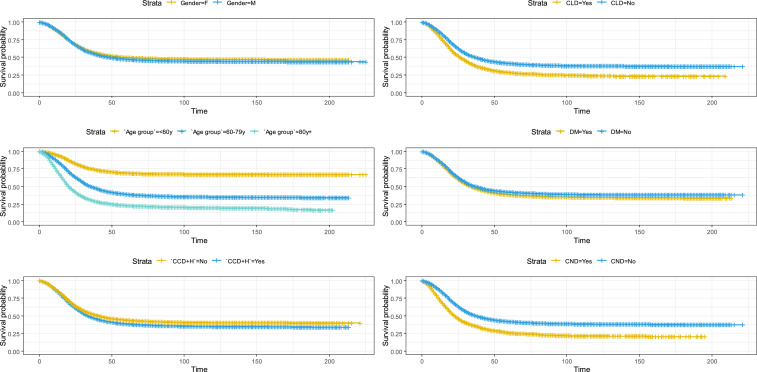
Kaplan-Meir plots for risk factors associated with death for coronavirus disease (COVID-19) patients. This figure appears in color at www.ajtmh.org.

Associations among sex, age group, four other prevalent comorbidities (chronic cardiovascular disease plus hypertension, chronic lung disease, diabetes mellitus, and chronic neurological disease) and the risk of COVID-19-related death are shown in Table 2. The corresponding Kaplan-Meier graph is presented in Figure 1. Men were at higher risk for death than women (HR, 1.22; 95% CI, 1.18–1.25), and the number of deaths was slightly higher for men than for women (28.1% versus 26.4%).

**Table 2:** Cox multivariate regression analysis of epidemiological characteristics and comorbidities associated with death for COVID-19 patients

Variable	Univariate analysis		Multivariate analysis	
	HR	95% CI	HR	95% CI
Sex				
Female	1.00		1.00	
Male	1.07	1.04–1.09	1.22	1.18–1.25
Age group, y				
< 60	1.00		1.00	
60–79	3.22	3.13–3.31	2.20	2.11–2.28
≥ 80	6.04	5.86–6.22	3.85	3.68–4.02
CCD + H				
No	1.00		1.00	
Yes	1.23	1.20–1.23	1.05	1.02–1.08
CLD				
No	1.00		1.00	
Yes	1.58	1.51–1.65	1.38	1.31–1.45
DM				
No	1.00		1.00	
Yes	1.14	1.11–1.17	1.14	1.11–1.18
CND				
No	1.00		1.00	
Yes	1.94	1.86–2.02	1.48	1.41–1.55

CCD + H = chronic cardiovascular disease plus hypertension; CLD = chronic lung disease; DM = diabetes mellitus; CND = chronic neurological disease. Source: Influenza Epidemiological Surveillance System (SIVEP-GRIPE) Epidemiological Surveillance Center of the State Health Secretary of São Paulo (CVE/SES-SP).

The rates of death among races (white, black, yellow, mixed, and indigenous) were similar, ranging from 21.8% to 33.5%. When the groups were separated into white and non-white (blacks and other minority ethnicities), the death rates were similar (white: 29.0% [17.902 of 61.659]; non-white: 29.0% [9.102 of 31.355]).

Increasing age was strongly associated with the risk of death, with patients 80 years or older having a more than three-fold increased risk compared with patients younger than 60 years (hazard ratio [HR], 3.85; 95% confidence interval [CI]], 3.68–4.02). The death rates among the younger than 30 years (6.1%; 358 of 5,879) and 30 to 59 years (13.6%; 7,278 of 53,368) age groups were considerably lower than that of the 60 years or older (41.3%; 25,415 of 61,475) age group, again highlighting the possible relationship of old age and death (Table 1).

The four most prevalent comorbidities, chronic cardiovascular disease plus hypertension (HR, 1.05; 95% CI, 1.02–1.08), chronic lung disease (HR, 1.38; 95% CI, 1.31–1.45), diabetes mellitus (HR, 1.14; 95% CI, 1.11–1.18), and chronic neurological disease (HR, 1.48; 95% CI, 1.41–1.55), were associated with an increased risk of death. As can be observed in Table 1, almost half of the patients who had chronic lung disease and chronic neurological disease died. Approximately half of the patients who needed care in the ICU died (48.5%; 20,220).

## DISCUSSION

The state of São Paulo is the most populous state in Brazil, with approximately 46 million inhabitants, and it is one of the main protagonists of the COVID-19 pandemic in Brazil. This study showed that a large number of laboratories confirmed that COVID-19 cases led to hospitalization in the state (120,804; incidence of 294 hospitalizations per 100,000 inhabitants), and that approximately one-third of cases of those hospitalized evolved into serious illness with the need for ICU care (34.5%) and death (27.4%). These results serve as an alert to the government and the population to recognize the need for urgent measures to control the pandemic.

Our findings revealed that men are at higher risk for death than women. These data are similar to those of recent studies that reported that SARS-CoV-2 infection was more likely to affect males.^4,5,10^ Therefore, male sex is a well-established risk factor for serious COVID-19 outcomes.

The results also showed that the majority of hospitalized patients were white, consistent with the first report of cases among people who had traveled internationally^11^; however, the numbers of deaths among races were similar when analyzed individually and equal when compared between whites and non-whites (blacks and other minority ethnicities). Non-white race was previously found to be associated with a higher risk of COVID-19 infection, risk of ICU admission, and death.^12,13^ This topic needs to be studied further because it is related to other characteristics, such as cultural and socioeconomic factors. Although there was no difference in the number of deaths among the non-white population in this study, there is an information gap for the race variable because this information only started to be collected more than 1 month after the beginning of the pandemic in Brazil, thus showing the disregard for racial vulnerability in the country during the pandemic.

Increasing risks of death were observed with increasing age, and advanced age is also a well-established risk factor for serious COVID-19 outcomes; these results were widely observed during other studies performed in several countries worldwides.^4,5,14,15^ Although the death rates among young people are considerably lower than those among elderly individuals, it is very important to discuss the roles of these people in maintaining the disease. In general, young people are more resistant to maintaining social isolation and safety measures to contain the spread of the virus, and they can also be a vital source of transmission for individuals at increased risk for serious outcomes of COVID-19.

Several studies have shown that individuals with chronic diseases are at higher risk for serious outcomes of COVID-19.^15,16^ Our findings support this information. Chronic cardiovascular disease plus hypertension, diabetes mellitus, chronic lung disease, and chronic neurological disease had a greater relationship with death, suggesting that these comorbidities may be associated with an increased risk of more serious outcomes among patients hospitalized with COVID-19.

The high death rate for patients admitted to the ICU observed during in this study was expected because of the critical condition of patients who are referred to this treatment unit because they require respiratory support and intensive care. However, other associated factors need to be studied further to determine if this high death rate is related to the severity of the illness also or also to the treatment methods used in the ICU.

There is a need for immediate measures to prevent and control the pandemic, especially for groups at higher risk for serious outcomes, so there will be fewer hospitalizations and deaths. Decreased mobility, social distancing, and the implementation of biosafety measures, such as wearing a mask, are essential for reducing the spread of the virus until vaccination coverage is sufficient to prevent high transmission. Another important factor is the government’s attention to the socioeconomically vulnerable population, which is predominantly black. This population has greater difficulty maintaining social isolation and has less access to the health system.

Our investigation of risk factors for deaths in São Paulo state had several limitations. Data extraction from hospitalized SARS-COV-2-positive patients in São Paulo occurred on October 10, 2020. The frequency and distribution of underlying conditions after that period may have changed as additional data became available. Many patients were still hospitalized at the time of data extraction. The notifications in the SIVEP-GRIPE system are detailed and longitudinal, but a considerably large amount of data were missing data, which may have impacted these results and introduced important information bias. Furthermore, some deaths related to COVID-19 may have been mistakenly classified as non-COVID-19, particularly during the beginning of the pandemic. However, it is possible that this inaccuracy has decreased rapidly as the number of deaths has increased.

## CONCLUSIONS

In São Paulo state, men, elderly individuals, and patients with chronic cardiovascular disease, including hypertension, chronic lung disease, diabetes mellitus, and chronic neurological disease, are at higher risk for death from COVID-19.
